# Comparative diagnosis of the alveolar antral artery canal in the lateral maxillary sinus wall in corresponding panoramic radiography and cone-beam computed tomography

**DOI:** 10.1186/s40729-023-00497-9

**Published:** 2023-09-19

**Authors:** Ali-Reza Ketabi, Stefan Hassfeld, Hans-Christoph Lauer, Andree Piwowarczyk

**Affiliations:** 1 Department of Prosthodontics, School of Dentistry, Faculty of Health, Witten/Herdecke University, Alfred-Herrhausen-Straße 45, 58455 Witten, Germany; 2 Department of Oral and Maxillofacial Surgery, Dortmund Hospital GmbH and Faculty of Health, Witten/Herdecke University, Muensterstr. 240, 44145 Dortmund, Germany; 3 Department of Prosthodontics, Center for Dentistry and Oral Medicine (Carolinum), Goethe-University, Theodor-Stern-Kai 7, 60596 Frankfurt, Germany; 4 Department of Prosthodontics, School of Dentistry, Faculty of Health, Witten/Herdecke University, Alfred-Herrhausen-Straße 45, 58455 Witten, Germany; 5 Private Dental Office of Dr Ali-Reza Ketabi, Epplestraße 29 a, 70597 Stuttgart, Germany

**Keywords:** Maxillary sinus, Sinus lift, CBCT, Diagnostic imaging, Panoramic radiography, Alveolar antral artery

## Abstract

**Purpose:**

Sinus lift operations are a tried and tested means of providing adequate implant prosthetics to patients with compromised jawbones. Knowledge of the arterial supply of the maxillary sinus region is essential for surgical treatment in this area. The aim of the present comparative study was to determine whether alveolar antral artery (AAA) canal can be diagnosed both in corresponding panoramic radiography (PR) and cone-beam computed tomography (CBCT).

**Methods:**

A total of 335 patients with 635 sites and corresponding maxillary sinus in both PR and CBCT were selected and examined for AAA canal visibility.

**Results:**

The visibility of the AAA canal was significantly higher in CBCT than in PR. A total of 154 (46.0%) AAA canals could be identified in the maxillary sinus on the right. However, only four (1.2%) of these were also visible in PR. The detected values of the AAA canals in the maxillary sinus on the left in the PR and CBCT images were similar to those of the right. While 164 AAA canals (49%) were observed in CBCT images, only 1 (0.3%) was identifiable in PR.

**Conclusions:**

The results show that CBCT can be recommended for visualising the AAA canal when surgically planning sinus augmentation procedures.

**Graphical Abstract:**

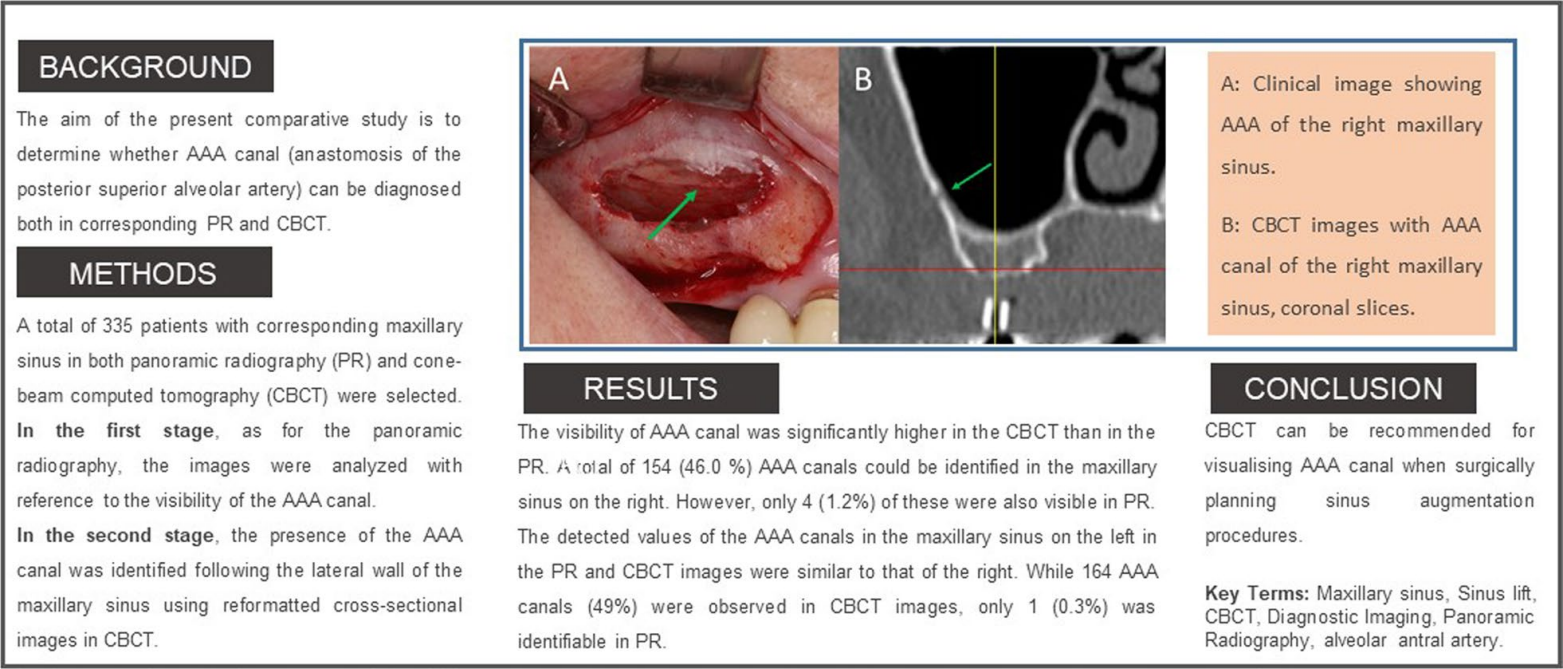

## Introduction

A compromised alveolar ridge and maxillary sinus are the primary limiting factors that make reconstruction of the posterior maxilla more challenging [1]. For restoring lost teeth with implant-supported dentures in optimal prosthetic positions, the sinus lift operation is currently crucial in dental surgery. Maxillary sinus elevation is regarded as an effective and predictable approach for augmenting the posterior maxilla, providing practitioners with adequate bone volume for implant placement [2–4].

**Fig. 1:**
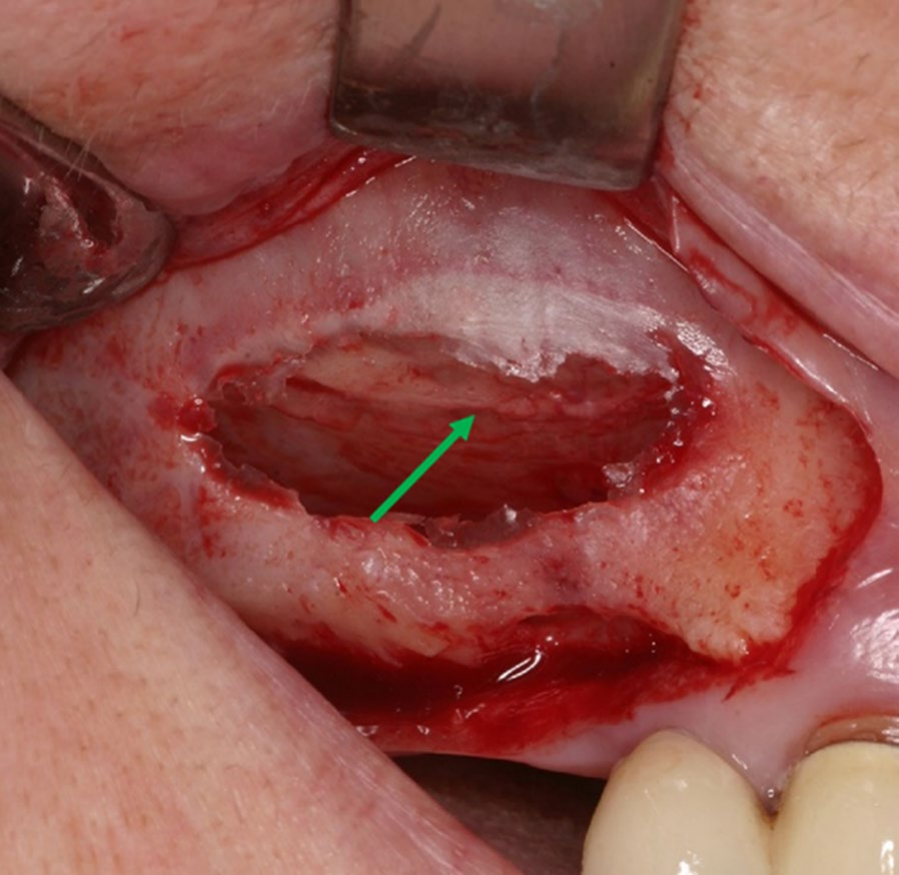
Clinical image showing AAA of the right maxillary sinus

The lateral wall and crestal approaches constitute the primary techniques for maxillary sinus augmentation. The lateral wall technique was first delineated by Tatum and subsequently, Boyne and James in 1980 [5, 6]. While the method is predictable and has high success rates, various complications have been documented during either surgery or the postoperative period [7]. One such complication is blood–vessel trauma, which may lead to severe haemorrhaging [8]. Accidental bleeding following surgical damage to the alveolar antral artery (AAA) is one of the two most frequent complications of sinus lifting (SL), along with perforation of the sinus membrane [9]. The AAA (Fig. 1) is an anastomosis of the posterior superior alveolar artery (PSAA) and the infraorbital artery (IOA), as has been repeatedly described in the literature [10, 11].

In a cadaveric study, Rosano et al. examined the arterial blood supply of the maxillary sinus to better understand the development of vascular complications that may result from surgical interventions in this region. The AAA was found in 100% of cases. The study determined that the AAA provides blood supply to both the sinus membrane and periosteal tissue and more specifically to the anterior lateral wall of the sinus. Therefore, detailed knowledge of the vascularization of the maxillary sinus is essential for the avoidance of vascular complications during surgical interventions in this region [12].

For the purposes of reducing such complications, it is recommended that a thorough radiological assessment of the maxillary sinus be carried out in the sinus region prior to surgical intervention [13, 14]. Both panoramic radiography (PR) and cone-beam computed tomography (CBCT) are described as advantageous as key diagnostic tools at the diagnostic stage and for general preoperative planning [15, 16]. Previous studies reported results based on small-scale samples, but comparative studies on detection accuracy of CBCT and PR remain limited. The aim of the present study was to provide a comparative evaluation of the diagnostic value of two-dimensional and three-dimensional radiographs through the recognizability of the AAA canal in the lateral wall of the maxillary sinus.

The hypothesis was that the AAA channel is identifiable in CBCT scans more often than in PR scans.

## Methods

Ethical approval was issued by the Committee of the Baden–Württemberg Medical Association (F-2014-006-z). The study was carried out in accordance with the ethical standards of the 1964 Declaration of Helsinki [17]. The present analysis was designed as a retrospective study. Between 2010 and 2017, patients who underwent both PR and a CBCT scan were selected from the database of a private practice in Stuttgart.

First, 549 patient records were selected and anonymized. The selection criteria were the availability of patients with corresponding maxillary sinus visible in both PR and CBCT images without artefacts in the measurement area, as well as correct patient positioning. A total of 335 patients with 635 sites fulfilled the selection criteria, of which 173 were female and 162 were male. The average ages were 62.1 years among female patients and 58.4 years among male patients.

The Panoramic Radiography used in this study were recorded by means of the Orthophos D 3297 X-ray unit (Sirona dental Systems GmbH, Bensheim, Germany) and saved on an imaging plate (Vistascan View, Dürr Dental, Bietigheim-Bissingen, Germany). The exposure parameters were set up at a tube voltage of 60 kV, a current of 10 mA and an exposure time of 16.4 s. These were read out with an imaging plate scanner (Vistascan Combi Plus, Dürr Dental). The evaluation was conducted by means of the radiographic software DBSWin (version 5.1.1; Dürr Dental SE, Bietigheim-Bissingen, Germany).

The CBCT images were recorded using a Gendex CBX-500™ (KaVo Dental GmbH, Biberach, Germany). The acquisition parameters were a tube voltage of 90 kV, an exposure time of 8.9 s with a 0.3 mm resolution, using a field of view (FOV) of 6 or 14 cm (diameter) and 5–8.5 cm (height). The images were evaluated with the i-cat Viewer software (Imaging Sciences International, Hatfield, PA, USA).

The substantiating indications for the radiographs were obtained independently of the study. All images were taken by an expert in dental radiography. The examiner had the requisite qualifications and competence for CBCT and was briefed by an expert in the field of dental radiography and CBCT prior to the beginning of the study. To verify the reliability of the radiographic measurements and evaluations, multiple assessments were performed on 20 randomly selected patients.

A reliability analysis (Cohen’s kappa coefficient) was conducted in a darkened room (< 1000 lx) using an accredited diagnostic monitor (EIZO FlexScan S2000 1024 × 1280 pixels) according to the radiographic instructions and under standardized conditions. Measurements were taken over a maximum of 6 h per day, including a 30-min break every 2 h. Inter-rater reliability of the outcomes between the examiner and expert was established. Furthermore, all images were examined a second time by the same examiner following an interval of 2 weeks for the calculation of intra-rater reliability. For both intra-observer reliability and inter-observer reliability, a kappa coefficient was computed.

**Fig. 2:**
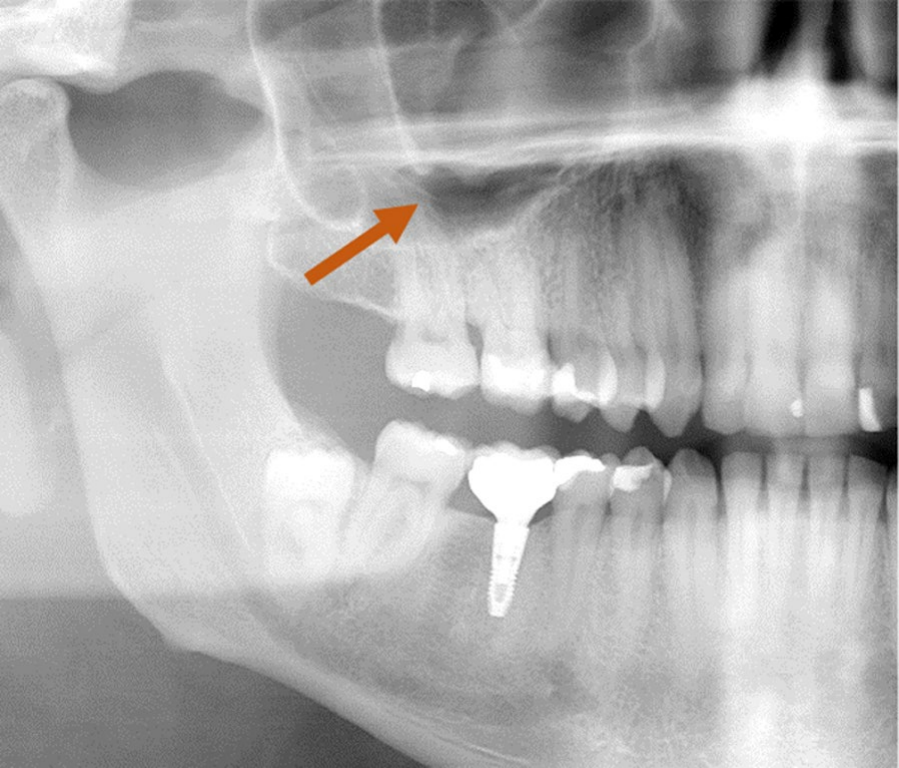
PR image with AAA canal of the right maxillary sinus

**Fig. 3:**
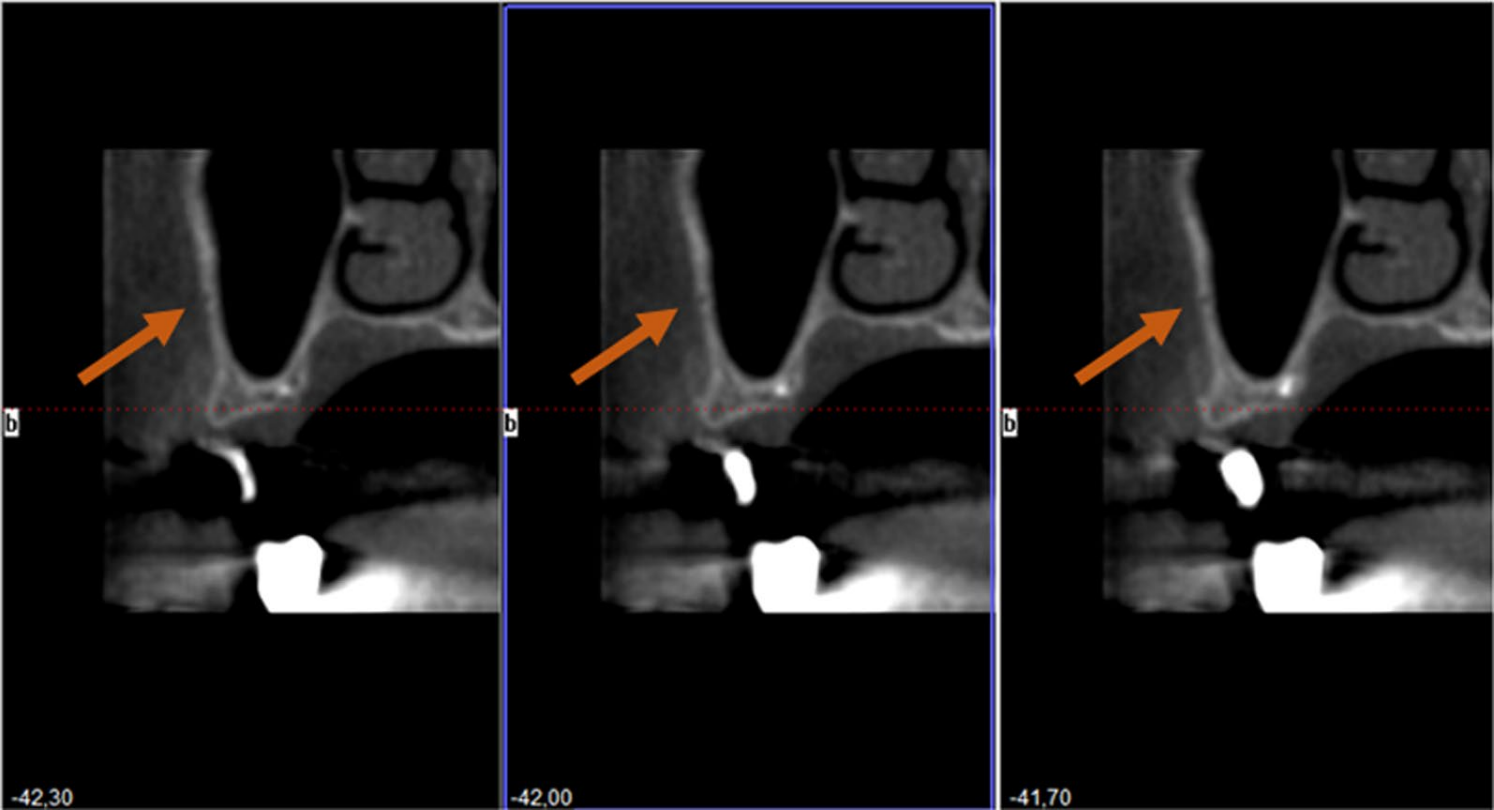
CBCT images with AAA canal of the right maxillary sinus, coronal slices

**Fig. 4:**
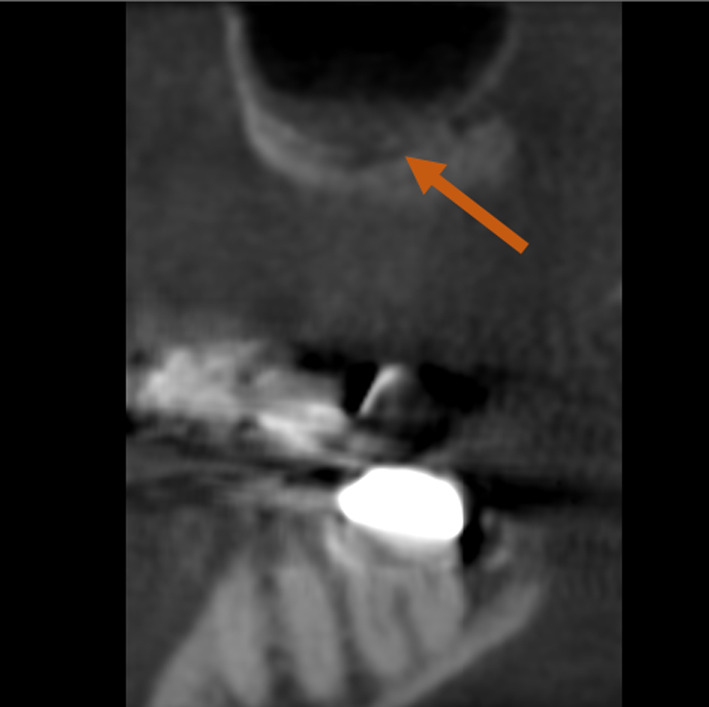
CBCT images with AAA canal of the right maxillary sinus, sagittal slice

Patient data were anonymised, and the radiographic images were numbered. A chart was used to process the patient data and the radiographic indications in Microsoft Excel 2016 (Microsoft Inc., Redmond, Washington, USA). Both the PR images (Fig. 2) and CBCT images (Fig. 3) were evaluated with respect to the detectable presence of the AAA canal in the lateral wall of the maxillary sinus on both the left and right. For the PR, the images were analysed with reference to the visibility of the AAA canal (Fig. 2). In the case of the CBCT images, the presence of the AAA canal was identified following the lateral wall of the maxillary sinus using reformatted cross-sectional images. Only when the intra-osseous canal was visible on cross-sectional images was it considered present (Figs. 3, 4).

The first step was to diagnose the PR images. This was followed by diagnostic analysis of the corresponding regions in the CBCT images. The findings were fed into a mask that had been specially developed by the Institute for Statistics (MediStat GmbH, Kornshagen, Germany) before being analysed by Mrs. Ulrike von Hehn (MediStat GmbH, Kronshagen, Germany) using the software SPSS Statistics 25 (IBM Corporation, Armonk, New York, USA). The results in this study had an explorative and descriptive character. Therefore, all results were expressed as absolute values (quantitatively with the mean and standard deviation) and incidence values (percentage).

## Results

For the present study, 549 patients were initially selected from the database of a dental practice in Stuttgart, Germany, after undergoing PR and CBCT between February 2010 and January 2017. Both a panoramic image and a CBCT image were already available prior to the commencement of the study. There were 335 patients (with 635 sites) who met the inclusion criteria. Thus, the cohort of 335 patients comprised a total of 173 female and 162 male patients. The average age of female patients was 62.1 years, and the average age of male patients was 58.4 years.

The mapping of jaw sections in the CBCT images and PR images was audited prior to the definitive evaluation. For many patients, the field of view (FOV) in the CBCT image was smaller than in the PR, so it was not possible to account for all sinuses in these patients. Tables 1 and 2 show the visibility of the AAA canal in the PR and in CBCT images, respectively. Missing areas in either CBCT or PR images (5.1% of the right and 5.4% of the left maxillary sinus) have been noted and are listed in Tables 3 and 4. However, if at least one maxillary sinus was visible in the images, these patients were not entirely removed from the study.

**Table 1:** Visibility of the AAA canal in PR in the lateral wall of the left and right maxillary sinus in per cent

		Quantity Left side/right side	% of the total Left side/right side	% valid cases
PR maxilla : AAA canal visible	No	339/334	68.8/67.7	99.7/98.2
	Yes	1/6	0.2/1.2	0.3/1.8
	Total	340/340	69.0/69.0	100.0
	System error	153/153	31.0/31.0	
Total		493	100.0

**Table 2:** Visibility of the AAA canal in CBCT in the lateral wall of the left and right maxillary sinus in per cent

		Quantity left side/right side	% of the total left side/right side	% valid cases
CBCT maxilla: AAA canal visible	No	162/174	32.9/5.3	45.9/49.3
	Yes	172/160	34.9/32.5	48.7/45.3
	Non-evaluable	19/19	3.9/3.9	5.4/5.4
	Total	353/353	71.6/71.6	100.0
	System error	140/140	28.4/28.4	
Total		493	100.0

**Table 3:** Contingency table of the AAA canal in the lateral wall of the right maxillary sinus in per cent

			PR: visibility of AAA canal on the right side		Total
			No	Yes	
CBCT: visibility of AAA canal on the right side	No	Quantity	163	1	164
		% of the total	48.7%	0.3%	49.0%
	Yes	Quantity	150	4	154
		% of the total	44.8%	1.2%	46.0%
	Not within the field of view	Quantity	16	1	17
		% of the total	4.8%	0.3%	5.1%
Total		Quantity	329	6	335
		% of the total	98.2%	1.8%	100.0%

**Table 4:** Contingency table of the AAA canal in the lateral wall of the left maxillary sinus in per cent

			PR: visibility of AAA canal on the left side		Total
			no	yes	
CBCT: visibility of AAA canal on the left side	No	Quantity	153	0	153
		% of the total	45.7%	0.0%	45.7%
	Yes	Quantity	163	1	164
		% of the total	48.7%	0.3%	49.0%
	Not within the field of view	Quantity	18	0	18
		% of the total	5.4%	0.0%	5.4%
Total		Quantity	334	1	335
		% of the total	99.7%	0.3%	100.0%

For both intra-observer reliability and inter-observer reliability, Cohen's kappa coefficient was computed as 1.0 with a 95% confidence interval of [0.92; 1.00]. Table 1 shows the visibility of the AAA canal in the lateral wall of the maxillary sinus in the PR images. Here, it can be seen that in the most PR images, no AAA canal was recognizable. The AAA canal of the wall of the maxillary sinus was visible in only one PR image on the left side (0.3%) and in 6 on the right side (1.8%) (Table 1). Table 2 shows the visibility of the AAA canal in the lateral wall of the maxillary sinus in the CBCT images. From the analysis of the CBCT images, the AAA canal was visible in 172 CBCT images on the left side (48.7%) and in 160 images on the right side (45.3%) of the wall of the maxillary sinus (Table 2).

Tables 3 and 4 provide detailed information on the AAA canal visibility in PR versus CBCT in the right and left maxillary sinus. Table 3 details the visibility of the AAA canal in the maxillary sinus on the right in the PR and CBCT images. Initially, visibility was significantly higher in CBCT than PR (Table 3). A total of 154 (46.0%) AAA canals could be identified. However, only 4 (1.2%) of these were also visible in PR. In 163 (48.7%) cases, the AAA canal could not be detected in either PR or CBCT. One (0.3%) of the detected AAA canals in PR was not visible in CBCT.

Table 4 shows the detected values of the AAA canal in the maxillary sinus on the left in the PR and CBCT images. As was the case on the right side, the AAA canal on the left side was also significantly more common in CBCT than in PR (Table 4). In the analysed CBCT images, 164 AAA canals (49%) were observed, while only 1 (0.3%) could be recognized in PR. There was no case in which the AAA canal was visible in PR but not CBCT.

**Fig. 5:**
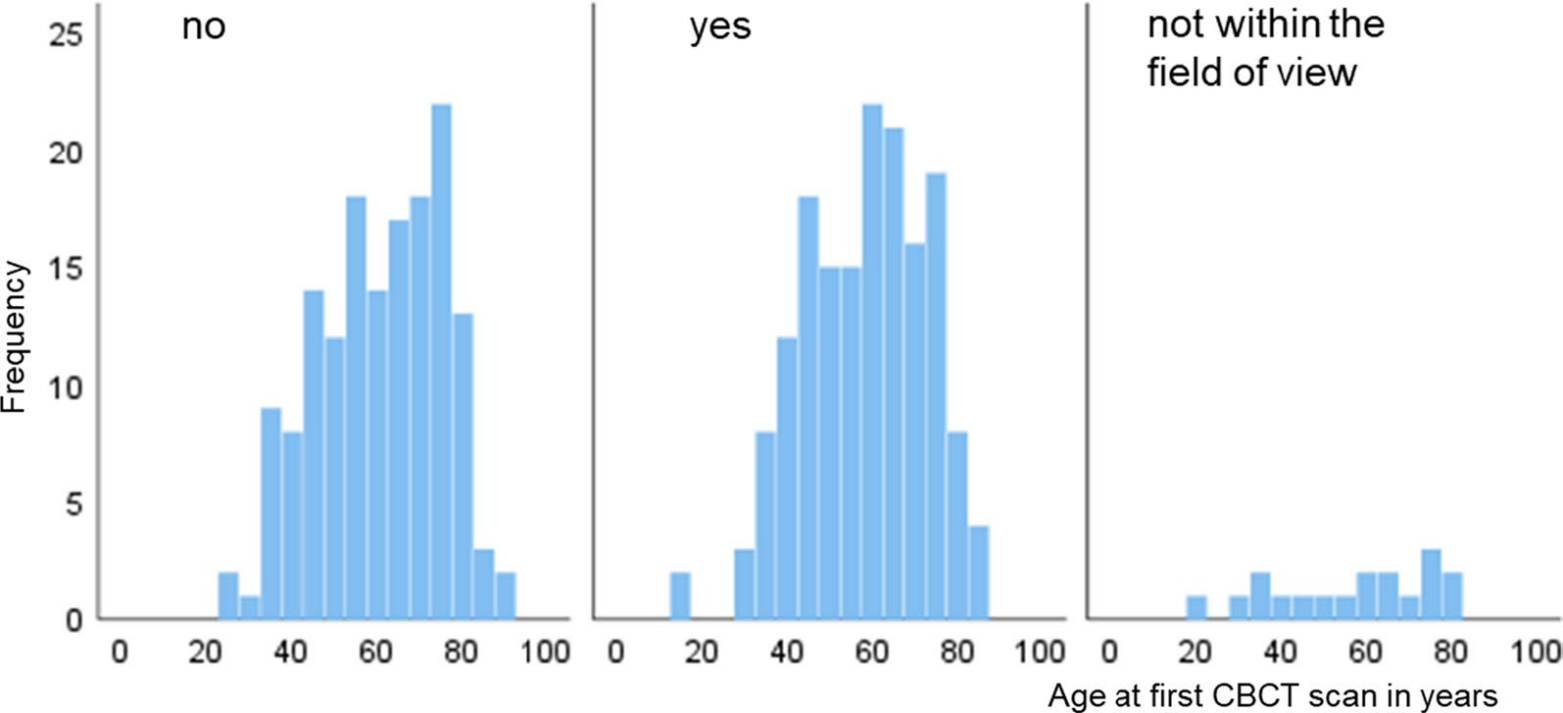
Distribution of patient age and visibility of the AAA canal in CBCT in the lateral wall of the maxillary sinus

Figure 5 shows the distribution of the patient's age and the visibility of the AAA canal in the lateral maxillary sinus wall of the CBCT images. The age distribution in the patient group with visible AAA canal is similar to the group in which AAA canal is not visible. There was no statistical significance (*p* > 0.05).

## Discussion

The aim of the present comparative study was to determine whether it is possible to diagnose the AAA canal in the lateral maxillary sinus wall in both corresponding PR and CBCT images. The hypothesis was that the AAA canal is more often identifiable in CBCT scans than in PR scans. The findings of our study indicate that in the CBCT images, the AAA canal could be detected in 154 (46%) cases in the maxillary sinus on the right. However, only 4 (1.2%) of these were also visible in PR (Table 3). One (0.3%) of the diagnosed AAA canals in PR could not be confirmed in CBCT.

A direct comparison of the corresponding images revealed that this may be caused by translucencies, such as those due to the hard palate or by imaging the dorsum of the tongue. Both of these are superimposed on the maxillary sinus in PR images and could result in misdiagnoses in the intervening translucent areas. From the available CBCT images, it was possible to detect 164 AAA canals (49%) on the left, while only 1 (0.3%) was recognizable in PR (Table 4). There was no case in which the AAA canal was visible in PR but not CBCT on the left.

No significant correlation was found between the presence of the AAA canal and age (*p* > 0.05).

As a first step, 549 patient records compiled in the period between 2010 and 2017 in a private dental practice in Stuttgart were selected and anonymized. A total of 335 patients (with 635 sites) fulfilled the selection criteria, of which 173 were female and 162 were male. The average age of the female patients was 62.1 years, and the average age of the male patients and 58.4 years. However, in contrast to similar comparative studies, the remaining 335 patients (with 635 sites) represent a high number of admissions used for statistical analysis [15, 18, 19].

In the preliminary stages, measurement integrity was verified by an expert. The measurements were repeated at 2-week intervals. For both intra-observer reliability and inter-observer reliability, Cohen’s kappa coefficient was computed as 1.0 with a 95% confidence interval of [0.92; 1.00]. Thus, a high degree of concordance was obtained.

Sinus lifting has become a common surgical intervention for increasing alveolar bone height prior to dental implant placement in the posterior maxilla [20]. However, specific complications must be accounted for intraoperatively, such as Schneiderian membrane perforation or bleeding from the antral alveolar artery [7, 9]. Maridati et al. reported that accidental bleeding of the AAA is one of the two most frequent complications of sinus lifting, along with perforation of the sinus membrane [9]. The AAA maintains a varying relationship with the sinus wall and is usually completely intraosseous. Only in rare instances (< 8%), it is more superficial on the lateral wall [21].

The detection of the AAA prior to dental procedures in connection with the maxillary sinus, such as elevation of the maxillary sinus floor, has been proven crucial for the avoidance of complications [7, 22]. Large-diameter blood vessels may impose more serious risk of bleeding during surgery [22]. Specialist literature recommends both PR and CBCT for diagnosis and planning prior to dental surgery [15, 16]. PR is a widely available, useful, and essential diagnostic tool in dentistry with respect to both diagnosis and general preoperative planning [23]. In PR, not every area of interest is accurately detected and allocated. The size and distribution of anatomical structures and lesions in the maxillary sinus affect visibility in PR. Furthermore, small maxillary sinus lesions (retention cysts, polyps, etc.) with a diameter of less than 3 mm show poor detection rates [18]. Particularly, CBCT leaves little room for interpretation of the findings and thus enables an examiner-independent assessment of specific findings that may be relevant for planned subsequent surgical interventions [24].

Three-dimensional imaging has been shown to be effective in the maxilla for a wide range of clinical settings, such as trauma, bone pathology, and neoplastic diseases, as well as dental implantology and sinus augmentation [24, 25]. In a systematic review of the assessment of the prevalence of an intraosseous canal in the lateral sinus wall, Varela et al. discovered that the detection of the canal has proven more frequent in CBCT studies (78.12%) than in CT studies (51.19%) [26]. They determined that in contrast to CBCT, conventional CT showed thicker arteries with a thickness of 0.5 mm or more. Thus, CBCT seems to provide reliable results with respect to the detectability of vascular canals. CBCT is a valuable diagnostic tool. Most previous studies recommend it as a presurgical evaluation of the maxillary sinus when identifying anatomical structures, particularly vascular supply [13, 27, 28].

In cadaver studies, both Rosano et al. and Sato et al. examined the prevalence of the AAA and discovered that it could be detected by dissection in 100% of the lateral sinus walls [12, 29]. By contrast, Temmerman et al. examined visibility in the lateral sinus wall and discovered AAA canals in 50% of the analysed CT images [30]. Likewise, Elian et al. and Mardinger et al. came to similar conclusions [31, 32]. As Mardinger et al. argue, a significantly lower number of detected AAA canals shows that the vessel must be of sufficient size to be identified by a CT scan.

The results of our study show similar observations regarding the visibility of the AAA canal and are confirmed by previous studies. The AAA canal could be observed in almost half of the CBCT images. It is possible smaller AAA canals were not visible in our CBCT images.

In addition to anatomical variations in the maxillary sinus, Shiki et al. evaluated pathological findings such as mucosal thickening, fluid retention, and sinus opacification related to the occurrence of maxillary sinusitis in PR and CBCT. They reported that soft tissues of the maxillary sinus cannot be effectively visualized in panoramic radiographs. One key result of this study was that the incidence of maxillary sinusitis was twice as high in patients opting for implant-supported restoration than in patients who did not [18].

They also found that some lesions in the maxillary sinus often have no symptoms initially. They concluded that the diagnosis of the pathological findings is often carried out incidentally when images of the area are obtained for other purposes. The authors recommend searching for these, since they are related to limitations in inserting dental implants and are causes of severe post-surgery inflammation. Above all, if the operation is unsuccessful, a worsening of the lesions would be expected [18].

Dau et al. discussed an entirely different aspect. In their experimental and comparative diagnostic study, they sought to determine whether the use of PR as opposed to CBCT impacts the evaluation of symptomatic maxillary sinus pathologies. Depending on the clinical and radiological experience of the observer, they found that PR alone remained insufficient for evaluating pathologies in the maxillary sinus [33]. This raises an interesting question of whether the presence of pathological findings influences the visibility of AAA canal in CBCT, meaning that in these cases, CBCT would not be indicated to detect AAA canals. Anamali et al. concluded that CBCT images provide highly instructive information, including the presence of AAA canal, regardless of the presence of intrasinusal pathoses [34].

Whether there is a correlation between the patient's age and the visibility of the AAA canal is controversial. While some working groups describe a correlation in their studies [32, 36], others could not find any correlation [37, 38].

In our study no significant association was found between the prevalence of the visibility of the AAA canal and age of participants. Further studies will be necessary here to be able to make a reliable statement.

The results of our study are consistent with other studies [24, 27, 34]. Accordingly, the present study shows that PR systematically underestimates the visibility of AAA canal. This was shown by directly comparing corresponding PR and CBCT images. Based on the present data, cross-sectional imaging may be recommended during the surgical planning of sinus augmentation procedures in visualising AAA canal for minimizing both intra- and postoperative complications.

Therefore, our hypothesis that there is a difference in the visibility of the AAA canal in the lateral maxillary sinus wall in favor of CBCT images compared to PR images can be agreed.

## Conclusion

The present study revealed the superiority of CBCT over PR. In contrast to PR images, the visibility of AAA canal is significantly higher in CBCT images. Therefore, for diagnosis of AAA canal in everyday practice, it makes sense to prioritize CBCT at least initially. Yet as a method, PR is not discarded, particularly in simple cases of dentistry. With respect to surgical planning in implantology, however, CBCT has proven a useful addition: it provides necessary information for avoiding complications early on in the perioperative planning phase.

The occurrence of anatomical variations and deviations from the norm should be accounted for during diagnosis and treatment planning phases. In recent years, the use of CBCT has become increasingly practicable and popular. Given that this procedure involves increased ionising radiation in comparison with PR, CBCT should only be done in cases in which the potential patient benefits outweigh the risks. Nonetheless, ethical and radiobiological aspects must be accounted for in accordance with the ALADA principle (“as low as diagnostically acceptable”) [35]. To establish more precise criteria for the preparation of CBCT in implant planning, further studies are essential. Aspects of surgery, prosthetics, and forensics should be accounted for.

## Data Availability

The original data sets analysed in the current study are available on reasonable request from Dr. Ali Reza Ketabi.

## Abbreviations

CBCT: Cone-Beam Computed Tomography
PR: Panoramic Radiography
AAA canal: Alveolar Antral Artery Canal
SL: Sinus Lifting
IOA: Infraorbital Artery
sec: Second
kV: Kilo Volt
mA: Milli Ampere
mm: Millimetre
cm: Centimetre
field of view: FOV
standard deviation: SD
